# Geriatric day surgery: challenge or opportunity?

**DOI:** 10.1186/1471-2482-13-S1-A24

**Published:** 2013-09-16

**Authors:** Carlo Grifasi, Massimiliano Petrocelli, Francesca Di Capua, Giuseppe Spinosa, Concetta Dodaro

**Affiliations:** 1 Department of Advanced Biomedical Sciences Federico II University, via S. Pansini 5, Naples, Italy

## Introduction

Given the recent advances in anesthesia, surgery, and monitoring technology, day surgery (DS) offers potential advantages for elderly patients undergoing elective surgery.

Epidemiological data show a continuous expansion of elderly population, associated with an increased demand for surgical treatments by older patients.

This review summarizes current selection criteria, anaesthesiology techniques and methods for perioperative management in geriatric outpatient surgery.

## Methods

The Medline database was searched using the terms: “Health needs in an aging global society”, “Day surgery and elderly patients”, “Elective vs emergency surgery”, “Challenges and opportunities of day surgery”, “Advances in anesthesiology and surgical techniques“, “Perioperative management in day surgery“, “Reducing waiting lists”.

Only papers in the English language from 1990 to 2012 were reviewed with a predominantly focus.

## Results

The key strengths of geriatric day surgery are: First of all, the reduced impact on the patient and family. Again, given the devastating effects perioperative infections play in elderly surgical patients, the reduced risk of hospital infections is especially beneficial in the case of reduced or compromised immune defence, a common pattern in elderly patients. Moreover, the brevity of hospital stay promotes early reassumption of active mobility, also reducing the risk of loss of autonomy. Finally, some evidence does exist in the literature that performing a given operation as DS is accompanied by reduced risk of postoperative disorders in comparison with prolonged hospitalization.

Scheduling elderly patients for elective day surgery instead of emergency surgery has proven to be safer.

Most of the DS procedures can be performed with minimally invasive anaesthesia, and are safely manageable on the day of the surgical operation.

There is general agreement that age as an independent risk factor for perioperative complications and death should not be considered as an exclusion criterion from surgery.

Comprehensive geriatric assessment (CGA) is currently used by geriatricians to evaluate the degree of frailty in elderly patients. Its use in preoperative assessment of surgical risk before cancer and noncancer surgery has been recently reported. It is foreseeable that such an approach will be extensively used in the future in preoperative evaluation of elderly patient.

Availability of caregivers can be lacking if the patient is older or lives alone. Social service availability varies considerably from one country to another.

Patient comprehension, can be altered or reduced due to sensorial deficits (visual, additive) or cognitive impairment.

As a principle, nor compensated, poorly stabilized patients should be treated with prolonged hospitalization, as they are at high risk of perioperative complications.

In general terms, all anaesthesia techniques, from local to general anaesthesia, may be applied. Randomized studies indicating which one should be considered the most suitable in elderly outpatients have not yet been published.

Statistically, the combination of sedation and local anaesthetics [monitored anaesthesia care (MAC)] is the most frequent anaesthesia technique in geriatric day surgery, due to the high number of elderly patients undergoing cataract, inguinal hernia repair and other day surgery procedures.

It has been demonstrated that postoperative pain after day surgery may last more than 3 days and affect quality of life for more than 7 days. Organizative aspects such as clear instructions at discharge, availability of analgesic drugs and follow-up are key factors, especially in geriatric day surgery.

## Conclusions

Geriatric day surgery may have a relevant social value, improving motion, functional autonomy and continence and reducing costs related to disability support.

Future research should focus on development of specific selection criteria, minimally invasive surgical techniques and effective and well tolerated postoperative pain treatment.
